# Predictors of Pressure Injuries in Older Residents Living in Nursing Homes in Sri Lanka: A Prospective Multi‐Site Cohort Study

**DOI:** 10.1111/jan.70036

**Published:** 2025-06-25

**Authors:** R. D. Udeshika Priyadarshani Sugathapala, Sharon Latimer, Brigid M. Gillespie, Aindralal Balasuriya, Wendy Chaboyer

**Affiliations:** ^1^ School of Nursing and Midwifery Griffith University, Gold Coast Campus Gold Coast Queensland Australia; ^2^ NHMRC Centre of Research Excellence in Wiser Wound Care Griffith University, Gold Coast Campus Gold Coast Queensland Australia; ^3^ Department of Nursing and Midwifery Faculty of Allied Health Sciences, General Sir John Kotelawala Defence University Colombo Sri Lanka; ^4^ Gold Coast University Hospital and Health Service Gold Coast Queensland Australia; ^5^ Department of Para Clinical Sciences, Faculty of Medicine General Sir John Kotelawala Defence University Colombo Sri Lanka

**Keywords:** nursing home care, older people, pressure injury, pressure ulcers

## Abstract

**Aim:**

To determine the predictors of pressure injuries among residents living in Sri Lankan nursing homes.

**Design:**

A prospective multi‐site longitudinal cohort study design.

**Methods:**

Semi‐structured observations and chart audits were used to gather data on 17 predictors of pressure injury from a consecutive sample of 210 residents (aged ≥ 60 years old) from nine nursing homes in Sri Lanka. Data were collected at baseline and followed up every week until the study endpoint: a new pressure injury or reaching the maximum 12 weeks of data collection, from July to October 2023. Validated semi‐structured data collection forms and chart audits were utilised. Binary logistic regression was used to identify the predictors of pressure injuries. Generalised linear mixed models were used to assess the association between predictors and the development of new pressure injuries.

**Results:**

The cumulative incidence of pressure injuries was 17.1% (36/210) during the 12 weeks. The number of medical devices and baseline pressure injuries predicted the development of new pressure injuries. Each additional medical device increased the likelihood of developing a pressure injury by 2.3‐fold, and individuals with a baseline pressure injury were 2.1 times more likely to develop a new pressure injury.

**Conclusion:**

Multiple medical devices and baseline pressure injuries are predictors of pressure injury in older residents living in nursing homes.

**Implications for the Profession:**

This study provides evidence of pressure injury predictors among older residents living in nursing homes. Early identification of high‐risk residents with an existing pressure injury and those with multiple medical devices is important for nurses and managers at nursing homes. Accurately assessing residents' risk of a pressure injury may result in implementing various preventive strategies that may ultimately help prevent future pressure injuries.

**Reporting Method:**

Strengthening the Reporting of Observational Studies in Epidemiology (STROBE) for cohort studies guidelines.

**Patient or Public Contribution:**

No patient or public contribution.

**Summary**

- This study highlights two easy‐to‐identify predictors of pressure injuries, specifically the number of medical devices and baseline pressure injuries among older residents living in nursing homes.
- These insights from Sri Lanka can inform and potentially refine global clinical guidelines and protocols for the prevention and management of pressure injuries to make them more applicable to both developed and developing countries.

## 1 Introduction

Health and well‐being in older residents aged 60 years or more are important globally. By 2050, this population will grow to about 1.5 billion or 16% of the global population (Padeiro et al. 2023). Despite initiatives such as the United Nation's decade of healthy ageing (2021–2030) (WHO 2024), some older residents who live with chronic illnesses require nursing home care (Jaul et al. 2018). These individuals are often at risk of developing a pressure injury (PI), defined as ‘localized damage to the skin and/or underlying soft tissue usually over a bony prominence or related to a medical or other device’ (NPIAP, EPUAP, PPPIA 2025). Meta‐analyses of 43 studies identified that the incidence and prevalence of PI in older residents living in nursing homes were 14.3% and 11.6%, respectively, with most occurring on the sacrum and heel (Sugathapala et al. 2023). Of the 43 studies, 19 countries were included in these analyses, which were undertaken in North and South America, Europe, Oceania and Asia; none were from developing countries. Further, there is limited evidence on factors associated with PI among older residents, particularly those in nursing homes located in developing countries where limitations on healthcare resources exist (Zhetmekova et al. 2024). This paper describes a study that determined predictors of PI among older residents living in nursing homes in Sri Lanka.

## 2 Background

PI causes severe pain (Mashouri et al. 2020), compromises defence mechanisms due to necrotic tissues, and increases the chance of local and systemic infection, particularly in stage III, IV, and unstageable cases (NPIAP, EPUAP, PPPIA 2025) and can result in recurrent hospitalisations (Cilla et al. 2023). Many factors that increase an individual's risk of PI development have been identified. The Coleman et al. (2014) conceptual framework categorises PI risk factors into two key components: (1) mechanical boundary conditions such as impaired mobility, inadequate or excessive skin moisture and sensory perception limitations; (2) susceptibility and individual tolerance, for example individual skin characteristics, skin temperature, nutritional status and blood biomarkers. Older age is also identified as a risk factor categorised into mechanical boundary conditions (EPUAP, NPIAP, PPPIA 2019). The skin safety model (SSM) describes the interplay between individual, external and environmental factors associated with various skin injuries, including PI in older residents in acute care settings (Campbell et al. 2016). It provides a theoretical framework for identifying potential predictors and their interaction with each other (Campbell et al. 2016). The model consists of four domains: (a) potential contributing factors to skin injury, (b) exacerbating elements, (c) potential skin injury and (d) potential outcomes of skin injury (Campbell et al. 2016). The SSM has also been applied to older residents in the community setting (Campbell and Samolyk 2020) and can guide clinicians and healthcare providers in recognising and considering the complexity of skin injury aetiology.

Several studies have examined the predictors of PI in older residents. A cohort study of older residents (aged ≥ 65 years) with limited mobility found that age, number of comorbidities and place of residence (aged care) were statistically significant PI predictors within the first 36 h of hospital admission (Latimer et al. 2019). Non‐blanchable erythema, a lower Braden Score and pressure area‐related pain have also been identified as independent risk factors of PI (Stage II–IV) in 308 high‐risk nursing home residents across 26 nursing homes in Belgium (Anrys et al. 2019). The authors of a systematic review and meta‐analysis noted that the majority of PI studies on older residents were conducted in two continents, Europe and North America (Sugathapala et al. 2023), thus only providing evidence on specific ethnic groups and skin types within other areas (Zhetmekova et al. 2024). There is limited representation of darker skin toned populations in this body of work to date. Variations in darker skin tones, individual demographics and health conditions in Sri Lankan older residents living in nursing homes enabled the identification of context‐specific predictors. Therefore, this study was important because, as a developing country, Sri Lanka has limited PI preventive resources (such as prophylactic dressings and high‐specification mattresses) and caregivers, which are contextually different from the most current available research evidence.

The high prevalence and severity of PI in nursing home residents highlight the urgent need for improved prevention strategies to enhance the quality of care and reduce associated healthcare costs. A better understanding of the unique PI predictors in older residents living in nursing homes will facilitate risk identification and allow more targeted preventive measures. Therefore, identifying predictors of PI can help nursing home staff recognise high‐risk residents and implement prevention strategies, improving care outcomes and reducing the incidence of PI. Understanding the predictors of PI among older residents with darker skin tones may be used to inform the development of a risk assessment tool specifically for this population and improve methods of early detection of PI. Studies of predictors of PI in developing countries are scarce; this is one of the first studies undertaken in Sri Lankan nursing home settings. Thus, this study addressed an essential gap in the literature.

## 3 Aim of the Study

This study aimed to determine the predictors of PI among Sri Lankan nursing home residents aged 60 years and older.

## 4 Methods

### 4.1 Study Design

A prospective multi‐site longitudinal cohort study design was used, with residents followed weekly for up to 12 weeks. The study is reported following the Strengthening the Reporting of Observational Studies in Epidemiology (STROBE) for cohort studies guidelines (von Elm et al. 2014).

### 4.2 Setting and Participants

Sri Lanka is a lower‐middle‐income country, and 12.3% of the population is aged 60 or older, making it the country with the highest proportion of older residents in South Asia (Solano 2021). The study was conducted in nine Sri Lankan nursing homes, known as elderly care homes. They are facilities designed to provide accommodation, healthcare and moderate assistance with daily activities for older residents who cannot live independently. Among the nine nursing homes, four were not‐for‐profit and five were for‐profit, with bed capacities ranging from 20 to 70. Most were in the Colombo district; three were from other districts. The admission procedure to a nursing home varies depending on each facility, but in general, the residents or guardians must apply for enrolment. Depending on the changes in residents' clinical conditions, they may transfer to other health care institutions, such as hospitals, or be discharged from the nursing home on family request. A consecutive sample of residents meeting the study selection criteria was invited to participate. The inclusion criteria were: (a) residents ≥ 60 years old, (b) with or without a PI (any stage) and (c) able to provide written consent independently or through their designated representatives at the time of recruitment.

As this study focuses on older residents, ≥ 60 years old was set as the age cutoff as per the definitions accepted by the UN (United Nations High Commissioner for Refugees 2018) and the WHO (World Health Organization 2017). Exclusion criteria were: (a) end‐of‐life care or (b) residents expected to be discharged. The nursing home managers/staff helped, excluding the residents receiving end‐of‐life care and residents expected to be discharged. In this study, it was anticipated that 10 PI predictors would be included in the final logistic regression model. For logistic regression modelling, 10 to 20 cases per PI predictor are required, with 20 cases being preferable (Polit 2010). Thus, a sample size of 200 residents was planned, but a larger sample helps achieve model stability (Polit 2010). Therefore, we aimed to recruit a sample of 200–210 residents. Written informed consent was obtained from participating residents or their legal representatives. Voluntary participation was emphasised during recruitment, and they were informed that they could withdraw from the study at any time during the study.

### 4.3 Outcome and Predictor Variables

The primary outcome or dependent variable was a new PI of any classification (stage I, II, III, IV, unstageable or deep tissue injury) based on the international clinical practice guidelines (EPUAP, NPIAP, PPPIA 2019). Four registered nurse research assistants, specifically trained for this study, identified pressure injuries through visual skin assessment.

**FIGURE 1:**
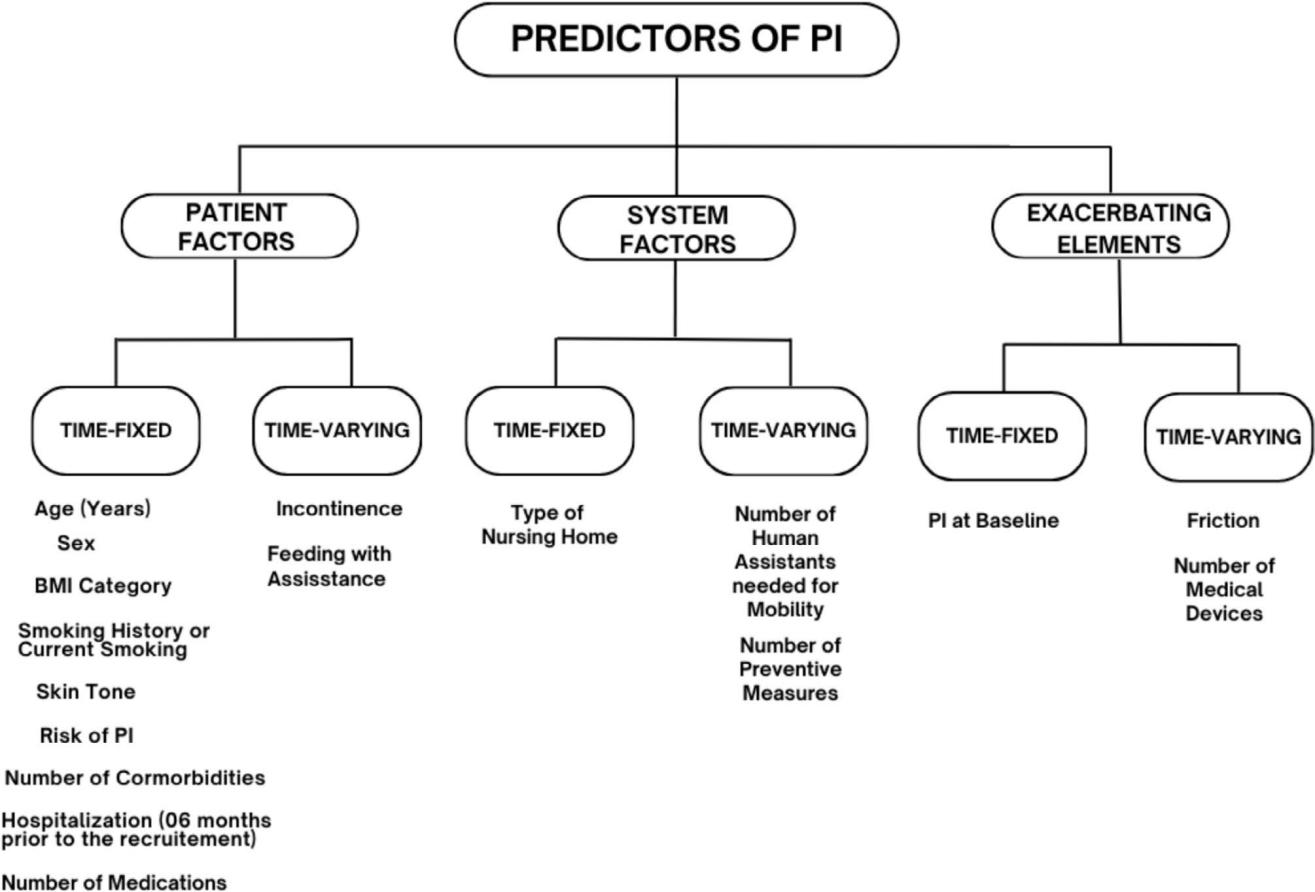
Potential predictors of PI based on the Skin Safety Model (Campbell et al. 2016).

The predictor variables were based on the SSM (Campbell et al. 2016) and previous empirical evidence (Coleman et al. 2014). The first two determinants, patient and system factors, are potential contributory factors, and the third determinant is exacerbating elements for skin injury. Predictors collected at baseline only were defined as time‐fixed variables, and those collected each week (or repeated measures) were defined as time‐varying variables, as shown in Figure 1. Data were collected on 17 predictor variables.

#### 4.3.1 Patient Factors

Patient factor variables were related to demographic characteristics, multiple comorbidities, mobility limitations, poor nutrition, incontinence, factors affecting perfusion and oxygenation, polypharmacy and alterations in sensation and cognition (Campbell et al. 2016). In this study, they included age (years), sex, body mass index (BMI) category (non‐healthy weight [BMI score < 18.5 kg/m^2^ or > 23 kg/m^2^]; healthy weight [BMI score: 18.5–23 kg/m^2^]) for Asian population (WHO Expert Consultation 2004), history of smoking or current smoking (yes; no), Fitzpatrick scale skin tone (light/moderate/brown; dark brown/black) (Fitzpatrick 1988), risk of PI (not‐at‐risk [Braden Score: 19–23]; at‐risk [Braden score: 18–6]) (Braden and Bergstrom 1987), number of comorbidities (‘none’, ‘one’, ‘two’, ‘three’, ‘four or five’), previous hospitalisations within last 6 months prior to the recruitment (January to July 2023), number of medications (‘none’, ‘one’, ‘two’, ‘three’, ‘four or above’), incontinence: urinary/faecal/dual (no and yes), feeding with assistance (oral feeding without assistance, oral feeding with assistant/nasogastric (NG) feeding/NG feeding supplemented with assisted oral feeding) and number of nurse assistants needed for mobility (‘none’, ‘one’, ‘two’, ‘three’, ‘four or above’).

#### 4.3.2 System Factors

System factors represented in the SSM denote the nursing home setting and comprise structural and process elements (Campbell et al. 2016). System structural elements can include clinical governance, safety culture, funding models, leadership, staffing and skill mix (Youngberg 2013). For this study, we included the type of nursing home setting based on funding (for‐profit/private, not‐for‐profit). The process elements in the SSM refer to interventions and activities that influence skin integrity outcomes (Campbell et al. 2016). We included the number of implemented preventive measures of PI each week (‘none,’ ‘one,’ ‘two,’ ‘three,’ ‘four or above’), such as preventive skin care, assessing nutritional status and repositioning.

#### 4.3.3 Exacerbating Elements

Exacerbating elements, the second domain in the SSM is conceptualised by the determinants pressure, shear, friction or the presence of irritants on the skin. Exposure to one or a combination of these determinants can result in skin injury (Campbell et al. 2016). For this study, we included baseline PI (yes; no), friction (yes; no) and the number of medical devices. PI can result from the use of devices designed and applied for diagnostic or therapeutic purposes, also known as medical devices (Arnold‐Long et al. 2017). Devices that are related to respiratory, orthopaedic, urinary/faecal collection, nasogastric and feeding tubes were considered under medical devices (EPUAP, NPIAP, PPIA 2019). We defined friction as occurring when the residents occasionally or frequently slid down in bed or a chair, and as no friction when they did not slide down in bed or a chair. Exacerbating elements were treated as independent factors in this study.

### 4.4 Data Collection

From July to October 2023, weekly semi‐structured observational and chart audit quantitative data were collected over 12 consecutive weeks by the first author and four trained Sri Lankan registered nurses. Previous PI incidence studies conducted in nursing homes used a 12‐week follow‐up period (Meesterberends et al. 2013; Van Gaal et al. 2014), and data were collected weekly for 12 weeks. Therefore, a period of 12 weeks was considered reasonable and feasible. Semi‐structured observational checklists were developed by the first author and validated by a panel of experts consisting of a wound care specialist, a wound care clinical nurse, and four wound care researchers. This panel was invited to comment on the relevancy, accuracy and appropriateness of the questions included in the data collection tools. Minor adjustments were made, such as removing ‘not applicable’ from some questions. After receiving ethics approval, the forms were pilot tested with 10 nursing home residents. Observation checklists were finalised without any changes. The data from the pilot test was not included in the study.

The baseline observation checklist consisted of demographic characteristics, results of the visual skin assessment, Fitzpatrick scale skin tone (Fitzpatrick 1988), Braden PI risk assessment (Braden and Bergstrom 1987), number and type of comorbidities, previous hospitalisation 6 months before the recruitment (January to July 2023), and type of medications. The weekly observational checklist included visual skin assessment, predictors and preventive measures of PI. Data were collected by directly speaking to residents or observing them or, when that was not feasible, by consulting with a nurse or nursing assistant and/or reviewing the resident's documentation. Study endpoints were the detection of a new PI, death, transfer to another facility or completing 12 weeks of follow‐up data collection.

Two weeks before data collection, the first author and an academic supervisor delivered a study‐specific training module to the four research assistants (Sugathapala et al. 2025). This included lectures, group discussion exercises and practical sessions covering PI risk factors and identification, staging for darker skin tones, Fitzpatrick skin tone assessment, Braden scale risk assessment, ethical considerations and an overview of data collection forms. Training materials were based on current clinical guidelines (EPUAP, NPIAP, PPPIA 2019) and literature on dark skin tone assessment (Dhoonmoon et al. 2023; Stankiewicz et al. 2016). The first author also trained the assistants on‐site, monitored their data collection and assessed inter‐rater reliability. The percentage of agreement on each observation checklist was calculated. Agreement percentages were 92%, 95%, 95% and 96%, with minor discrepancies resolved by consensus.

### 4.5 Data Analysis

Baseline data were analysed using descriptive statistics. Categorical variables were tabulated as frequencies/percentages, and continuous variables were presented as means and standard deviations (SD) for normally distributed data and median and interquartile range (IQR) for non‐normally distributed data. A generalised linear mixed model (GLMM), considering the person (resident) as the random effect, was used to examine the relationship between independent variables and PI while accounting for the repeated measures structure of the data. To develop the GLMM, a purposeful selection process of variables was performed. First, all 17 potential predictors were analysed using binary logistic regression analysis. Second, the variables with *p* ≤ 0.2 in the binary logistic regression analyses (Blay and Marchesoni 2011) were entered into the GLMM. However, due to the high number of predictor variables, not all were included in one GLMM, and they were grouped into three sets, and several GLMMs were created. The best fit model was selected by comparing the model parameters. GLMMs do not typically produce a single *p*‐value for the entire model like ANOVA or regression models. While reporting overall model significance is not always required, it can be useful to include model fit statistics to justify the appropriateness of the model. Common measures include: Akaike Information Criterion (AIC) or Bayesian Information Criterion (BIC) (Bolker et al. 2009). Sex and age were considered biologically important determinants; therefore, they were included in the first and subsequent models, regardless of their statistical significance levels (Cai et al. 2019; Elli et al. 2022). The following assumptions were checked prior to the regression analysis: sufficient outcome cases per predictor; multicollinearity between the independent variable predictors; and a visual inspection for outliers (Field 2013). Data were entered and analysed using IBM's Statistical Package for the Social Sciences (SPSS) version 29, following a thorough process of data cleaning and accuracy verification. The level of statistical significance was set at *p* < 0.05 and 95% confidence intervals were used where appropriate.

### 4.6 Ethics Statement

This study was approved by the Ethics Review Committee, Faculty of Medicine, General Sir John Kotelawala Defence University, Sri Lanka (RP/2023/09), and Griffith University Human Research Ethics Committee, Australia (2023/420). Written permission was also obtained from the nursing home managers to access the nine nursing homes.

## 5 Results

### 5.1 Sample Characteristics

Initially, 210 nursing home residents were recruited to the study, with 8 (3.8%) lost to follow‐up; one resident died, and the rest were discharged from the nursing home and, therefore, unable to follow up. Consequently, 202 residents remained at the study's endpoints (developing a new PI or completing the 12‐week follow‐up). Their average age was just over 77 years, and most were females (Table 1). There were 36 new patients who developed a new PI (i.e., cumulative incidence of 17.1%), of which 29 (80.6%) developed in females and 7 (19.4%) in males. There were 17 residents with PI at the baseline (PI prevalence 8.1%). Significant differences in nursing home setting (*p* = 0.025), risk of PI (*p* < 0.001) and baseline PI (*p* < 0.001) were observed between the residents with a new PI and those who did not develop a PI. That is, nursing home residents in the settings of for‐profit, those at risk of PI (Braden score: 18–6), and those with a baseline PI had more PIs at the end of the 12‐week follow‐up.

**TABLE 1:** Characteristics of residents with and without PI (*n* = 202).

Characteristics	Total sample	At study completion (PI or 12 weeks)		
		No PI	PI	*p*
	*n* (%)	*n* (%)	*n* (%)	
	202 (100)	166 (82.2)	36 (17.8)	—
Age (years)				
Median (IQR)	76.6 (72.1–82.9)	76.2 (72.7–81.9)	81.3 (68.7–87.3)	0.248^a^
Sex				
Female	163 (80.7)	134 (80.7)	29 (80.6)	0.982^b^
Male	39 (19.3)	32 (19.3)	7 (19.4)	
Setting				
For‐profit (*n* = 5)	112 (55.4)	86 (51.8)	26 (72.2)	0.025^b^
Not‐for‐profit (*n* = 4)	90 (44.6)	80 (48.2)	10 (27.8)	
BMI category				
Healthy weight (18.5–23 kg/m^2^)	68 (34.3)	55 (33.3)	13 (39.4)	0.503^b^
Non‐healthy weight (< 18.5 kg/m^2^ or > 23 kg/m^2^)	130 (65.7)	110 (66.7)	20 (60.6)	
Smoking history				
Ex or current smoker	10 (5.0)	7 (4.2)	3 (8.3)	0.302^b^
Non‐smoker	192 (95.0)	159 (95.8)	33 (91.7)	
Fitzpatrick skin tone				
Light/moderate brown	141 (69.8)	117 (70.5)	24 (66.7)	0.651^b^
Dark brown/black	61 (30.2)	49 (29.5)	12 (33.3)	
Braden risk of PI				
Not‐at‐risk (score: 19–23)	143 (70.8)	133 (80.1)	10 (27.8)	< 0.001^b^
At risk (score: 18–6)	59 (29.2)	33 (19.9)	26 (72.2)	
Baseline PI				
No	186 (92.1)	159 (95.8)	27 (75.0)	< 0.001^b^
Yes	16 (7.9)	7 (4.2)	9 (25.0)	
Previous hospitalisations				
Yes	171 (84.7)	143 (86.1)	28 (77.8)	0.207^b^
No	31 (15.3)	23 (13.9)	8 (22.2)	
Number of comorbidities				
0	38 (22.9)	5 (13.9)	43 (21.3)	0.790^c^
1	60 (36.1)	14 (38.9)	74 (36.6)	
2	36 (21.7)	10 (27.8)	46 (22.8)	
3	23 (13.9)	4 (11.1)	27 (13.4)	
≥ 4	9 (5.4)	3 (8.3)	12 (5.9)	
Number of medications				
0	49 (24.3)	43 (25.9)	6 (16.7)	0.409^c^
1	39 (19.3)	34 (20.5)	5 (13.9)	
2	42 (20.8)	30 (18.1)	12 (33.3)	
3	33 (16.3)	26 (15.7)	7 (19.4)	
≥ 4	39 (19.3)	33 (19.9)	6 (16.7)	

^a^ Mann–Whitney *U* test.

^b^ Chi‐squared test.

^c^ Fisher's exact test.

### 5.2 Predictors of PI Among Nursing Home Residents

Following binary logistic regression analysis, 10 variables met the *p* < 0.2 criteria (risk of PI, baseline PI, number of nurses needed to assist with mobility, number of medical devices, incontinence [urinary, faecal or dual], feeding with assistance, previous hospitalisations, friction, type of setting and number of preventive measures) (Table 2). A summary of time‐varying variables over the 12‐week data collection period is presented in Table S1.

**TABLE 2:** Predictors of PI (binary logistic regression).

Predictor	*β*	OR	95% CI		*p*
			LL	UL	
Age (years)	0.019	1.019	0.980	1.060	0.344
Sex (reference: male)	0.010	1.010	0.440	2.322	0.981
Setting (reference: for‐profit)	−0.830	0.436	0.209	0.908	**0.027**
BMI category (reference: healthy weight)	−0.267	0.765	0.379	1.548	0.457
History of smoking or current smoking (reference: no)	0.555	1.742	0.526	5.771	0.363
Skin tone (reference: light or moderate brown)	0.162	1.175	0.584	2.365	0.650
Risk of PI (reference: not‐at‐risk)	2.118	8.311	3.981	17.349	**< 0.001**
Baseline PI (reference: no)	1.845	6.327	2.905	13.783	**< 0.001**
Number of comorbidities	0.139	1.149	0.877	1.506	0.312
Previous hospitalisation: from January to July 2023 (reference: no)	0.521	1.684	0.760	3.727	**0.199**
Number of medications	0.090	1.095	0.923	1.298	0.300
Number of human assistants needed for mobility	1.217	3.377	2.458	4.640	**< 0.001**
Friction (reference: no)	0.729	2.072	0.721	5.953	**0.** **176**
Number of medical devices	1.713	5.545	3.580	8.587	**< 0.001**
Incontinence: urinary, faecal or dual (reference: no)	2.225	9.254	4.643	18.445	**< 0.001**
Assistance to feed (reference: no)	2.174	8.796	4.212	18.366	**< 0.001**
Number of preventive measures	−0.181	0.835	0.672	1.037	**0.** **103**

*Note:* Bold values indicate 10 variables meet the *p* < 0.2 criteria.

Abbreviations: CI, confidence interval; LL, lower limit; OR, odds ratio; UL, upper limit.

The outcome or dependent variable was the development of a new PI during the study. Other than age and sex, only three predictors, baseline PI, number of medical devices and number of preventive measures, were included in the final GLMM as fixed effects. The random effect was the resident in each nursing home. The model used a binomial distribution with a logit function. The AIC was 12,057.57, and the BIC was 12,063.26. The overall model was significant, *F* (5, 2191) = 5.26, *p* < 0.001, indicating the number of medical devices and the presence of baseline PI were significant predictors of developing a PI among the residents (Table 3). Based on the model, those with a baseline PI were 2.1 times more likely to develop a new PI (95% CI [1.03, 4.31], *p* = 0.04). When the number of medical devices increases by 1, the probability of PI occurrence increases by 2.3 times (95% CI [1.59, 3.35], *p* < 0.001). The random effects parameter was redundant, indicating no variability between the residents.

**TABLE 3:** Independent predictors of PI in older nursing home residents (GLMM).

Variable	Category	*β*	OR	95% CI for OR		*p*
				LL	UL	
Age (years)		−0.003	0.99	0.97	1.02	0.818
Sex	Female	0.03	1.03	0.59	1.80	0.893
	Male	Reference				
Baseline PI	Yes	0.75	2.1	1.03	4.31	**0.039**
	No	Reference				
Number of medical devices		0.84	2.3	1.59	3.35	**< 0.001**
Number of preventive measures		−0.04	0.96	0.82	1.11	0.552

*Note:* AIC (Akaike Information Criterion) = 12,057.57, BIC (Bayesian Information Criterion) = 12,063.26, Log‐Likelihood = −12,055.57. Bold values indicate *p* < 0.05.

Abbreviations: CI, confidence interval; LL, lower limit; OR, odds ratio; UL, upper limit.

## 6 Discussion

Guided by the SSM (Campbell et al. 2016), this prospective longitudinal cohort study determined the predictors of PI among nursing home residents in Sri Lanka. The cumulative incidence of 17.1% in this study is comparable to other recent studies. Two retrospective cohort studies reported an incidence of PI among nursing home residents as 13.1% (Akhtar‐Danesh et al. 2022) and 17.8/100 residents per year (Dhingra et al. 2023) from Canada and the United States, respectively. Residents who had a baseline PI and increasing numbers of medical devices in situ were significant predictors of PI (any stage) in this sample. These two predictors belong to the exacerbating elements in SSM (Campbell et al. 2016). Exacerbating elements are also known as ‘skin injury threats’ and exposure to one or a combination of these determinants can result in skin injury (Campbell et al. 2016). Even though the SSM focused on all skin injuries, this study focused on one injury, PI. To the best of our knowledge, this is the first study that identified predictors of PI based on the SSM and developed using previous literature on skin integrity (Campbell et al. 2016). Further, the data we collected on predictors was also informed by previous empirical evidence (Coleman et al. 2014). Because only two variables predicted pressure injury in this study, there is still more to be learned about PI risk factors in the nursing home setting.

We found individuals with a baseline PI were twice as likely to develop a new PI than those without a baseline PI. The potential impact of a previous PI on a new PI is also acknowledged in the international clinical practice guidelines' good practice statement (EPUAP, NPIAP, PPPIA 2019). Thus, it is important to find the underlying cause of an existing PI and implement an individualised management plan to prevent the development of another PI in the future. The results of our study suggest that with each additional medical device, the likelihood of developing a PI increases by twofold. Medical devices, like indwelling urinary catheters, are generally more rigid than the skin and the underlying soft tissues; thus, each one may compromise the skin if they are positioned in a way that increases pressure. These devices exert continuous forces and cause deformations to the skin and deeper tissues near their points of contact (Gefen et al. 2022). In the acute care setting, numerous studies have shown a relationship between mechanical devices and PI (Arnold‐Long et al. 2017; Brophy et al. 2021; Jackson et al. 2019). While hospitalised patients may differ from nursing home residents, the underlying mechanism of pressure on vulnerable tissue remains similar. Therefore, it is important to assess the underlying skin of the medical device to early detect the signs of PI (EPUAP, NPIAP, PPPIA 2019).

Even though we hypothesised 17 predictors of PI among nursing home residents, our confirmatory analysis showed that only the baseline PI and the number of medical devices would be ideal to use as predictors of PI, particularly for this sample. Although we derived possible predictors from SSM elements (Campbell et al. 2016); patient and system factors such as age, sex, BMI category, smoking history, skin tone, number of comorbidities, number of medications and number of preventive measures, these were not statistically significant predictors of PI according to our GLMM. These results were in contrast to Latimer et al. (2019), who found age, multiple comorbidities and type of residence (aged care) were significant predictors of PI in a sample of 1047 older residents with limited mobility. This could be due to the small sample size of our study compared to Latimer et al. (2019) study. In another cohort study of 2604 nursing home residents, female nursing home residents with advanced age, dementia and a history of cerebrovascular disease had a significantly higher risk of developing a PI (Norton scale score ≤ 9) (Elli et al. 2022).

### 6.1 Strengths and Limitations

Our study has several strengths as well as limitations. To determine the predictors of a PI (any stage), we collected prospective data weekly for 12 weeks from older residents with a minimal loss to follow‐up rate. Research assistants were registered nurses specifically trained to identify PI in darker skin toned individuals using visual skin assessment, and they could return to the setting multiple times to limit missing data. Several previous incidence studies conducted in nursing homes used a 12‐week follow‐up period (de Souza and de Gouveia Santos 2010; Meesterberends et al. 2013). We had a few selection criteria to recruit a representative sample of the target population. However, the results may not be generalisable to other nursing homes in the country. Although we used skin tone as a predictor of PI, it was not shown to be a significant predictor due to a lack of variation within the sample, with almost all residents having darker skin tones. This lack of variation in skin tone is also a limitation of our study.

### 6.2 Implications for Policy, Clinical Practice and Research

To our knowledge, this was the first study conducted in Sri Lanka that focused on pressure injury predictors among older residents. Developing and implementing policies that specifically address the care of residents who have had an existing PI and those with multiple medical devices would be ideal. The predictors, baseline PI and the number of medical devices in situ are readily available, cheap and easy to measure. They can be identified on admission during the nursing home resident's initial skin assessment and subsequent assessments. Thus, these measures can be used to better identify older residents at increased risk of PI. The findings of this research can be used to inform clinical practice in nursing home environments. Developing and implementing individualised management plans in collaboration with the resident, their family and/or carers, and the multidisciplinary team has been recommended as a key action for health service organisations (Australian Commission on Safety and Quality in Health Care 2020). Knowledge of the predictors of PI will enable nursing home staff to identify residents at high and low risk of PI and include them in prevention plans. To avoid the development of PI and its harmful sequela, residents should receive evidence‐based PI prevention and treatment care.

Nevertheless, nurses and carers should be aware of the management of medical devices and minimise their impact on the skin. For example, avoiding positioning the resident directly onto medical devices, such as urinary catheter tubing, also prevents constant pressure over the skin and prevents new PI (EPUAP, NPIAP, PPPIA 2019). Checking the placement of devices regularly and using protective skin barriers will reduce the damage caused by medical devices (Arnold‐Long et al. 2017). Where possible, alternative devices, such as non‐adhesive securement methods (bandages), should be used to hold the device in place, which are less likely to cause PI.

Policies and practice guidelines without active strategies to ensure that they are used are not likely to benefit the residents. Educating nursing home staff on the increased risk of PI in residents with these predictors can be done through establishing PI prevention teams, regular meetings and educational handouts or posters. Understanding the contextual factors, barriers and enablers of providing education, staff training and initiating PI prevention care bundles may aid in supporting knowledge translation and implementation of guidelines (Graham et al. 2006; Lynch et al. 2018). Consequently, they may allocate resources such as pressure‐relieving mattresses for high‐risk residents. Regular training sessions can help staff stay updated on best practices for prevention and care. Most importantly, further research on the incidence of PI will help evaluate whether preventive care has been effectively implemented. Future research should aim to develop and evaluate the effectiveness of tailored intervention programmes designed for residents with a history of pressure injuries and those with multiple medical devices.

## 7 Conclusion

This study aimed to gain a better understanding of predictors of PI among Sri Lankan nursing home residents. The study contributes to our understanding of the clinical application of SSM (Campbell et al. 2016), from which we selected the possible predictors. The presence of multiple medical devices and baseline PI emerged as important predictors of PI among this sample. If used to improve PI risk assessment, these findings have significant implications for the early detection of PI among nursing home residents in Sri Lanka. Protocols to prevent PI can be developed based on these results. This study is limited by the relatively small sample. Future research could assess the predictive ability of other predictors with a larger sample.

## Author Contributions

**R. D. Udeshika Priyadarshani Sugathapala:** writing – original draft, writing – review and editing, visualisation, methodology, investigation, formal analysis, conceptualization. **Sharon Latimer:** writing – review and editing, supervision, methodology, formal analysis, conceptualization. **Brigid M. Gillespie:** writing – review and editing, supervision, methodology, formal analysis, conceptualization. **Aindralal Balasuriya:** writing – review and editing, supervision, methodology, formal analysis, conceptualization. **Wendy Chaboyer:** writing – review and editing, supervision, methodology, formal analysis, conceptualization. All authors have made substantial contributions to the work, revised it critically, approved the final version and agreed to be accountable for its accuracy and integrity.

## Conflicts of Interest

The authors declare no conflicts of interest.

## Supporting information


Data S1.

**Table S1.** Summary of time‐varying variables over the 12 weeks of data collection.

## Data Availability

The data that support the findings of this study are available from the corresponding author upon reasonable request.
