# Supplementary material for: Predictors of Pressure Injuries in Older Residents Living in Nursing Homes in Sri Lanka: A Prospective Multi‐Site Cohort Study
Source: J Adv Nurs. 2025 Jun 25;82(4):3065–75. doi: 10.1111/jan.70036 (PMC12994681; doi:10.1111/jan.70036)
Supplement: Supplementary file 2 — Table S1. Summary of time‐varying variables over the 12 weeks of data collection. [file JAN-82-3065-s001.docx]

**Supplementary Materials**

**Table S1: Summary of time-varying variables over the twelve weeks of data collection**

| **Week (number of residents observed each week)** | **1**  **(n=210)**  **n (%)** | **2**  **(n=203)**  **n (%)** | **3**  **(n=199)**  **n (%)** | **4**  **(n=195)**  **n (%)** | **5**  **(n=187)**  **n (%)** | **6**  **(n=181) n (%)** | **7**  **(n=181)**  **n (%)** | **8**  **(n=172)**  **n (%)** | **9**  **(n=168)**  **n (%)** | **10**  **(n=168)**  **n (%)** | **11**  **(n=167)**  **n (%)** | **12**  **(n =166)**  **n (%)** |
| --- | --- | --- | --- | --- | --- | --- | --- | --- | --- | --- | --- | --- |
| **Incontinence: urinary/faeces/dual** | 31 (14.8) | 29 (14.3) | 30 (15.0) | 32  (16.4) | 28  (15.0) | 35  (19.3) | 41 (22.7) | 30  (17.4) | 32 (19.0) | 28  (16.7) | 27 (16.2) | 27  (16.2) |
| **Feeding with assistance** | 12  (5.7) | 11  (5.4) | 9  (4.5) | 9  (4.6) | 9  (4.8) | 9  (4.9) | 10  (5.5) | 9  (5.2) | 11  (6.5) | 8  (4.8) | 8  (4.8) | 8  (4.8) |
| **Friction** | 9  (4.3) | 5  (2.5) | 6  (3.0) | 9  (4.6) | 9  (4.8) | 13  (6.9) | 19  (10.5) | 16  (9.3) | 8  (4.8) | 13  (7.7) | 11  (6.6) | 9  (5.4) |
| **Number of nurse assistants needed for mobility** | | | | | | | | | | | | |
| None | 144  (68.6) | 149  (73.4) | 138  (69.3) | 139  (71.3) | 140  (74.9) | 132  (72.9) | 127  (70.2) | 127  (73.8) | 125  (74.4) | 128  (76.2) | 124  (74.3) | 126  (75.9) |
| One | 46  (21.9) | 31  (15.3) | 38  (19.1) | 36  (18.5) | 29  (15.5) | 32  (17.7) | 34  (18.8) | 33  (19.2) | 31  (18.5) | 28  (16.7) | 33  (19.7) | 34  (20.5) |
| Two | 13  (6.2) | 18  (8.9) | 19  (9.6) | 19  (9.7) | 16  (8.5) | 15  (8.3) | 18  (9.9) | 11  (6.4) | 8  (4.7) | 8  (4.7) | 9  (5.4) | 5  (3.0) |
| Three | 7  (3.3) | 5  (2.4) | 4  (2.0) | 1  (0.5) | 2  (1.1) | 2  (1.1) | 2  (1.1) | 1  (0.6) | 4  (2.4) | 4  (2.4) | 1  (0.6) | 1  (0.6) |
| **Number of medical devices** | | | | | | | | | | | | |
| None | 184  (87.6) | 173  (85.2) | 175  (88.0) | 167  (85.7) | 162  (86.6) | 150  (82.9) | 145  (80.1) | 139  (80.8) | 139  (82.7) | 141  (83.9) | 145  (86.8) | 144  (86.7) |
| One | 19  (9.0) | 23  (11.3) | 20  (10.0) | 24  (12.3) | 22  (11.8) | 30  (16.6) | 35  (19.4) | 32  (18.6) | 29  (17.3) | 26  (15.5) | 22  (13.2) | 22  (13.3) |
| Two | 6  (2.9) | 6  (3.0) | 3  (1.5) | 3  (1.5) | 3  (1.6) | 1  (0.5) | 1  (0.5) | 1  (0.6) | 0  (0.0) | 1  (0.6) | 0  (0.0) | 0  (0.0) |
| Three | 1  (0.5) | 1  (0.5) | 1  (0.5) | 1  (0.5) | 0  (0.0) | 0  (0.0) | 0  (0.0) | 0  (0.0) | 0  (0.0) | 0  (0.0) | 0  (0.0) | 0  (0.0) |
| **Number of PI preventive strategies implemented** | | | | | | | | | | | | |
| One | 0  (0.0) | 0  (0.0) | 0  (0.0) | 0  (0.0) | 0  (0.0) | 1  (0.5) | 0  (0.0) | 1  (0.6) | 1  (0.6) | 0  (0.0) | 2  (1.2) | 1  (0.6) |
| Two | 0  (0.0) | 1  (0.5) | 2  (1.0) | 1  (0.5) | 0  (0.0) | 0  (0.0) | 2  (1.1) | 1  (0.6) | 0  (0.0) | 1  (0.6) | 0  (0.0) | 1  (0.6) |
| Three | 0  (0.0) | 2  (1.0) | 2  (1.0) | 1  (0.5) | 5  (2.7) | 6  (3.3) | 7  (3.9) | 6  (3.5) | 6  (3.6) | 6  (3.6) | 5  (3.0) | 4  (2.4) |
| Four | 11  (5.2) | 5  (2.5) | 10  (5.0) | 13  (6.7) | 14  (7.5) | 18  (9.9) | 19  (10.5) | 19  (11.0) | 15  (8.9) | 15  (8.9) | 17  (10.2) | 11  (6.6) |
| Five | 19  (9.1) | 16  (7.9) | 24  (12.1) | 29  (14.9) | 25  (13.4) | 32  (17.7) | 17  (9.4) | 23  (13.4) | 19  (11.3) | 24  (14.3) | 17  (10.2) | 27  (16.3) |
| Six | 36  (17.1) | 29  (14.3) | 34  (17.1) | 52  (26.7) | 42  (22.4) | 45  (24.9) | 59  (32.6) | 55  (32.0) | 43  (25.6) | 43  (25.6) | 31  (18.5) | 29  (17.5) |
| Seven | 85  (40.5) | 88  (43.2) | 71  (35.7) | 65  (33.3) | 70  (37.4) | 51  (28.2) | 50  (27.6) | 44  (25.6) | 45  (26.8) | 41  (24.4) | 48  (28.7) | 47  (28.4) |
| Eight | 44  (21.0) | 46  (22.7) | 53  (26.6) | 29  (14.8) | 27  (14.4) | 26  (14.4) | 20  (11.0) | 16  (9.3) | 28  (16.7) | 28  (16.7) | 40  (24.0) | 40  (24.0) |
| Nine | 15  (7.1) | 16  (7.9) | 3  (1.5) | 5  (2.6) | 4  (2.2) | 2  (1.1) | 7  (3.9) | 7  (4.0) | 11  (6.5) | 10  (5.9) | 7  (4.2) | 6  (3.6) |
